# Sintilimab plus R-CHOP versus DA-EPOCH-R as first-line therapy for primary mediastinal large B-cell lymphoma: a real-world comparative cohort study

**DOI:** 10.3389/fimmu.2026.1924360

**Published:** 2026-10-13

**Authors:** Zhihe Liu, Jianxin Ren, Kaiqin Jiang, Hao Guan, Xia Zhao

**Affiliations:** 1 Department of Lymphoma, the Affiliated Hospital of Qingdao University, Qingdao, China; 2 Department of Oncology, Gaomi People’s Hospital, Weifang, China; 3 Department of Hematology, Linyi Central Hospital, Linyi, China

**Keywords:** PD1, PD-L1, primary mediastinal large B-cell lymphoma, R-CHOP, sintilimab

## Abstract

**Background:**

Primary mediastinal large B-cell lymphoma (PMBCL) is a highly curable lymphoma in which first-line therapy must balance durable disease control with acute and late treatment toxicity.

**Methods:**

We retrospectively analyzed 98 patients with newly diagnosed PMBCL who received sintilimab plus R-CHOP (group 1, n = 42) or DA-EPOCH-R (group 2, n = 56). The primary endpoint was progression-free survival (PFS), and secondary endpoints included complete metabolic response (CMR) and safety. Propensity-score matching (PSM) and inverse probability of treatment weighting (IPTW) were used to adjust for baseline imbalances. Molecular profiling was performed in 75 diagnostic samples to characterize immune-escape features, including 9p24.1 alterations and PD-L1/PD-L2 expression.

**Results:**

Sintilimab plus R-CHOP produced numerically comparable response and PFS outcomes to DA-EPOCH-R, although equivalence or non-inferiority cannot be inferred from this retrospective, event-limited cohort. CMR rates were 85.7% versus 83.9% before matching (Fisher P = 1.000) and 83.3% versus 80.6% after PSM (P = 1.000; 36 pairs). Estimated 24-, 36-, and 60-month PFS rates were 81.0% versus 83.9% before matching (HR 1.19, 95% CI 0.46–3.09; log-rank P = 0.716), 83.3% versus 80.6% after matching (HR 0.84, 95% CI 0.28–2.51; P = 0.759), and 83.9% versus 84.6% after IPTW (HR 1.04, 95% CI 0.39–2.76; robust P = 0.942), respectively. These estimates were based on 17 PFS events in the full cohort and 13 events after matching. Grade 3–4 neutropenia was less frequent with sintilimab plus R-CHOP before matching (38.1% versus 66.1%; P = 0.008) and remained lower after matching (30.6% versus 61.1%; P = 0.017). In 75 profiled diagnostic samples, group-level molecular data confirmed the expected immune-escape architecture of PMBCL and showed broadly similar distributions of 9p24.1 alterations and PD-L1/PD-L2 expression between treatment groups.

**Conclusion:**

In this real-world cohort, sintilimab plus R-CHOP produced overlapping response and PFS estimates relative to DA-EPOCH-R, together with less grade 3–4 neutropenia. The study cannot establish equivalence, non-inferiority, treatment replacement, or radiotherapy omission. These findings support the biological rationale for integrating PD-1 blockade into frontline chemoimmunotherapy and highlight the need for prospective studies to define its role within PET-adapted and immune-driven treatment strategies for PMBCL.

## Introduction

Primary mediastinal large B-cell lymphoma is a distinct clinicopathological entity that arises from thymic B cells and most often affects adolescents and young adults, with a female predominance and a characteristic anterior mediastinal presentation (1–5). Contemporary classifications and disease-focused reviews emphasize that PMBCL is biologically distinct from diffuse large B-cell lymphoma, not otherwise specified, and shares selected features with classical Hodgkin lymphoma (1–5). These shared features include frequent 9p24.1 copy-number alteration, JAK-STAT pathway activation and PD-L1/PD-L2 overexpression, which together provide a strong rationale for studying immune-checkpoint blockade in this disease (6–11).

Frontline therapy for PMBCL remains an area of active debate. DA-EPOCH-R is widely used because it can achieve high cure rates and reduce historical reliance on mediastinal radiotherapy (12–15). However, this regimen is logistically intensive and is associated with clinically meaningful myelosuppression, infection risk and treatment burden (12–15). R-CHOP-based approaches remain common in routine practice, but they are often paired with radiotherapy or PET-adapted consolidation strategies, creating uncertainty about the optimal balance between disease control, treatment intensity and late toxicity (4, 16–19).

Randomized and prospective evaluation of PD-1 blockade in untreated PMBCL is underway. ANHL1931 (NCT04759586) is a phase 3 trial of nivolumab added to physician-selected DA-EPOCH-R or R-CHOP with or without radiotherapy (20). ALLG-PACIFIC (NHL35) is a phase 2 study of pembrolizumab plus R-CHOP, including a rituximab–pembrolizumab window before chemotherapy (21). Published frontline results for PD-1 plus chemoimmunotherapy remain scarce, which motivated the present real-world comparison.

Post-treatment assessment is especially challenging in PMBCL because residual mediastinal masses are common and may reflect fibrosis or inflammatory change rather than viable lymphoma. PET-based CMR therefore has a central role in response assessment, particularly when residual anatomical abnormalities persist (17, 22–24). The randomized IELSG37 trial strengthened the evidence base for omitting radiotherapy in patients who achieve CMR after induction immunochemotherapy, but its findings do not remove the need for careful PET-based selection or prospective validation in new treatment contexts (17, 18). This issue is particularly relevant for PD-1-containing regimens, in which immune activation may complicate image interpretation while also strengthening the rationale for metabolic rather than anatomical decision-making (9–11, 22–24).

Against this background, we compared sintilimab plus R-CHOP with DA-EPOCH-R in a real-world cohort of patients with newly diagnosed PMBCL. We also integrated descriptive gene-expression and copy-number analyses to assess whether the cohort showed the expected molecular architecture of PMBCL and whether immune-escape features were broadly balanced between treatment groups (6–8, 25, 26). The central question was whether sintilimab plus R-CHOP could yield overlapping disease-control estimates while reducing hematologic toxicity and informing exploratory PET-directed post-treatment management. The study was not designed to establish equivalence, non-inferiority, treatment replacement or radiotherapy omission.

## Methods

### Study design and patients

This retrospective cohort study included patients with newly diagnosed PMBCL recorded in our institutional clinical databases between January 2019 and December 2024. Baseline characteristics, immunophenotype, treatment exposure, PET response, residual mass, adverse events and PFS were extracted from the clinical database. The data cutoff for censored follow-up was June 2026. The study was approved by the medical Ethics Committee of the Affiliated Hospital of Qingdao University (No. QYFY WZLL 50345). Written informed consent was obtained from all patients, and the study was conducted in accordance with the Declaration of Helsinki. Patients were treated at three hospitals. During the study window, both sintilimab plus R-CHOP and DA-EPOCH-R were available at each site; regimen choice was made by the treating physician after discussion with the patient rather than by a center-restricted protocol. Accordingly, no hospital used only one of the two strategies.

### Treatment

The sintilimab plus R-CHOP regimen consisted of sintilimab 200 mg on day 1, rituximab 375 mg/m^2^ on day 2 and CHOP chemotherapy on day 3, repeated every 21 days for six planned cycles. CHOP comprised cyclophosphamide 750 mg/m^2^, pirarubicin 40 mg/m² (substituted for conventional doxorubicin), vincristine 1.4 mg/m^2^ (maximum, 2 mg) and prednisone 100 mg daily for 5 days. DA-EPOCH-R was administered for six planned cycles according to the dose-adjusted infusional platform reported by Dunleavy et al. (12). In this institutional adaptation, doxorubicin was replaced by pirarubicin given as a continuous infusion at 8 mg/m²/day on days 1–4 (32 mg/m² per cycle), together with etoposide, vincristine, cyclophosphamide, prednisone, rituximab and filgrastim. Subsequent cycle doses of etoposide, pirarubicin and cyclophosphamide were adjusted according to the nadir absolute neutrophil count and platelet count using the published pharmacodynamic algorithm (12). Among 56 patients treated with DA-EPOCH-R, the dose level was escalated in 37 patients (66.1%), left unchanged in 11 (19.6%) and reduced in 8 (14.3%). After completion of systemic therapy, patients with partial metabolic response received radiotherapy, whereas patients with CMR and residual mass were managed with observation.

### Response and safety assessment

Response was assessed by PET-based Deauville score according to the Lugano classification and international lymphoma imaging recommendations (22–24). The primary endpoint was PFS, defined as the interval from diagnosis to progression, relapse, death or last follow-up. Secondary endpoints were CMR, defined as Deauville score 1–3 at the end of treatment, and safety. Partial metabolic response was defined as Deauville score 4–5 with reduced uptake compared with baseline, and progressive metabolic disease was defined as new lesions or unequivocal progression. Follow-up for censored patients was updated to June 2026. Median follow-up was estimated by the reverse Kaplan-Meier method. Adverse events, with emphasis on grade 3–4 cytopenias, febrile neutropenia, infection and immune-related adverse events, were summarized for all patients.

### Gene-expression analysis

Gene-expression profiling was available for 75 diagnostic samples from the 98-patient clinical cohort, including 31 patients treated with sintilimab plus R-CHOP and 44 treated with DA-EPOCH-R according to the clinical treatment record. The molecular dataset contained curated mutation calls, categorical copy-number calls, transcript abundance reported as log2TPM and precomputed pathway scores. CNV amplification or loss and pathway-score interpretation followed established PMBCL molecular-profiling frameworks (25, 26). High pathway-score groups were defined relative to the cohort median for continuous pathway scores unless categorical classification had already been provided. Prespecified molecular comparisons between treatment groups included 9p24.1 amplification, PD-L1 and PD-L2 expression, JAK-STAT activation status and immune-escape classification. No molecular subgroup analysis of CMR or PFS was prespecified as a powered endpoint. A *post hoc* descriptive cross-tabulation of 9p24.1 status, treatment arm and PFS events is provided, and is not interpreted as a predictive biomarker result.

### Statistical analysis

Categorical outcomes were summarized as counts and percentages and compared using two-sided Fisher exact tests. Continuous variables were summarized as medians and ranges, or medians and interquartile ranges for molecular expression variables, and compared using Wilcoxon rank-sum tests. PFS at fixed time points was estimated with the Kaplan-Meier product-limit approach, with censoring marks displayed on survival curves, and compared using the log-rank test. Hazard ratios were estimated using Cox proportional-hazards models. Because treatment allocation was non-randomized, propensity scores were estimated with logistic regression using age, sex, ECOG performance status 0-1, Ann Arbor stage I-II, IPI 0-2, LDH elevation, bulky disease and superior vena cava syndrome. IPI was retained as the conventional composite risk score used in routine PMBCL care, accepting partial collinearity with stage, LDH and performance status. Baseline mass diameter, extranodal involvement, B symptoms and SUVmax were not entered into the propensity model; they were instead inspected after matching (**Table 1**) and examined in a tumor-burden-adjusted Cox sensitivity model. A 1:1 nearest-neighbour propensity-score matched analysis without replacement used a 0.2 SD caliper on the logit of the propensity score. Inverse probability of treatment weighting with stabilized weights truncated at the 1st and 99th percentiles was performed as a sensitivity analysis outside the matched cohort. Exploratory sensitivity analyses included tumor-burden-adjusted Cox models incorporating baseline mass, extranodal involvement and bulky disease, and treatment-by-IPI interaction testing. P values are descriptive and were not adjusted for multiplicity. All statistical analyses were performed using R 4.6.0 (R Foundation for Statistical Computing, Vienna, Austria) with survival, MatchIt, and cobalt.

**Table 1:** Baseline characteristics before and after propensity-score matching, with IPTW balance.

Characteristic	Group 1 (n=42)	Group 2 (n=56)	P	SMD	After PSM group 1 (n=36)	After PSM group 2 (n=36)	After PSM P	After PSM SMD	IPTW SMD
Age, median (range), years	32.0 (18.0-51.0)	35.0 (18.0-50.0)	0.118	-0.31	34.5 (18.0-51.0)	33.5 (18.0-50.0)	0.962	-0.01	0.03
Female sex, n (%)	30 (71.4%)	31 (55.4%)	0.141	0.34	24 (66.7%)	23 (63.9%)	1.000	0.06	-0.03
ECOG 0-1, n (%)	23 (54.8%)	35 (62.5%)	0.534	-0.16	21 (58.3%)	22 (61.1%)	1.000	-0.06	-0.06
Ann Arbor stage I-II, n (%)	27 (64.3%)	37 (66.1%)	1.000	-0.04	23 (63.9%)	22 (61.1%)	1.000	0.06	-0.00
IPI 0-2, n (%)	32 (76.2%)	36 (64.3%)	0.269	0.26	26 (72.2%)	25 (69.4%)	1.000	0.06	-0.02
B symptoms, n (%)	19 (45.2%)	31 (55.4%)	0.414	-0.20	16 (44.4%)	17 (47.2%)	1.000	-0.06	-0.19
Superior vena cava syndrome, n (%)	13 (31.0%)	17 (30.4%)	1.000	0.01	12 (33.3%)	14 (38.9%)	0.806	-0.12	0.03
Bulky disease >=7.5 cm, n (%)	35 (83.3%)	49 (87.5%)	0.574	-0.12	31 (86.1%)	32 (88.9%)	1.000	-0.08	0.00
Extranodal/extra-mediastinal involvement, n (%)	10 (23.8%)	17 (30.4%)	0.503	-0.15	8 (22.2%)	13 (36.1%)	0.300	-0.31	-0.27
LDH elevated, n (%)	33 (78.6%)	45 (80.4%)	1.000	-0.04	30 (83.3%)	31 (86.1%)	1.000	-0.08	-0.03
Beta-2 microglobulin elevated, n (%)	25 (59.5%)	31 (55.4%)	0.837	0.08	22 (61.1%)	17 (47.2%)	0.344	0.28	0.18
Baseline mass diameter, median (range), cm	10.0 (4.2-14.7)	10.4 (5.4-15.6)	0.102	-0.39	10.2 (5.8-14.7)	10.0 (5.4-15.6)	0.697	-0.12	-0.29
Baseline SUVmax, median (range)	17.9 (5.3-32.3)	20.2 (5.0-36.1)	0.125	-0.23	18.5 (5.3-32.3)	20.1 (7.1-36.1)	0.325	-0.18	-0.20

CI, confidence interval; ECOG, Eastern Cooperative Oncology Group; IPI, International Prognostic Index; IPTW, inverse probability of treatment weighting; LDH, lactate dehydrogenase; PSM, propensity-score matching; SMD, standardized mean difference.

## Results

### Patient characteristics

A total of 98 patients were included: 42 received sintilimab plus R-CHOP and 56 received DA-EPOCH-R. Baseline characteristics were broadly comparable between groups, although imbalances were observed in age, IPI and tumor burden (**Table 1**). Most patients presented with advanced-stage disease and high-risk features, including elevated LDH and bulky mass. After propensity-score matching, 36 patients were retained in each group. Balance improved for most prespecified covariates, although residual imbalance persisted for selected variables, including extranodal involvement and beta-2 microglobulin elevation. Inverse probability of treatment weighting provided an additional full-cohort sensitivity analysis.

### Response and progression-free survival

Before matching, objective response was achieved in 39 (92.9%) patients treated with sintilimab plus R-CHOP and 51 (91.1%) patients treated with DA-EPOCH-R. CMR (Deauville 1-3) was observed in 36 (85.7%) and 47 (83.9%) patients, respectively (P = 1.000; **Figure 1A**). After propensity-score matching, ORR was 91.7% versus 88.9%, and CMR was 83.3% versus 80.6% (P = 1.000; **Figure 1B**). With follow-up to June 2026, median follow-up was 53.3 months in the sintilimab plus R-CHOP group and 65.8 months in the DA-EPOCH-R group. Progression occurred in 8 (19.0%) and 9 (16.1%) patients, respectively, yielding 17 PFS events in the full cohort (Fisher P = 0.790). Median PFS was not reached in either group. Estimated PFS rates at 24, 36 and 60 months were 81.0% with sintilimab plus R-CHOP and 83.9% with DA-EPOCH-R (HR 1.19, 95% CI 0.46-3.09; log-rank P = 0.716; **Figure 2A**). In the matched cohort, 6 and 7 PFS events occurred in the sintilimab plus R-CHOP and DA-EPOCH-R groups, respectively. The corresponding estimated 24-, 36- and 60-month PFS rates were 83.3% versus 80.6% (HR 0.84, 95% CI 0.28-2.51; log-rank P = 0.759; **Figure 2B**). In the inverse probability-weighted Cox sensitivity analysis, the estimated PFS rates were 83.9% versus 84.6% (HR 1.04, 95% CI 0.39-2.76; robust P = 0.942; **Figure 2C**). Point estimates therefore overlap, but the analysis is event-limited: the confidence intervals are compatible with both a clinically important advantage and a clinically important disadvantage of either regimen. Equivalence or non-inferiority cannot be inferred.

**Figure 1:**
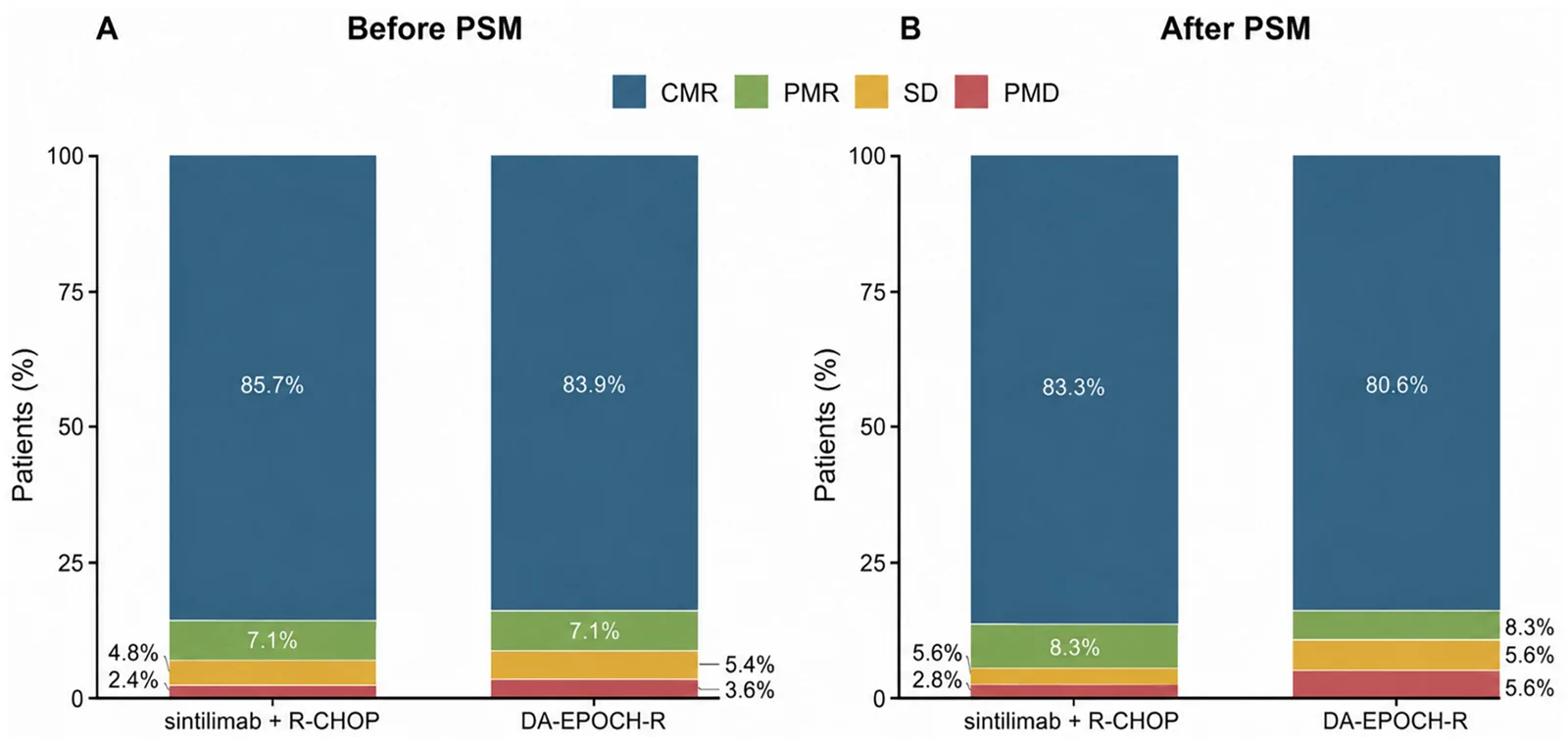
End-of-treatment metabolic response distribution before and after propensity-score matching. **(A)** Response distribution before matching. **(B)** Response distribution after propensity-score matching. Stacked bars show the proportions of patients achieving complete metabolic response (CMR; Deauville 1-3), partial metabolic response (PMR), stable disease (SD) or progressive metabolic disease (PMD) after sintilimab plus R-CHOP or DA-EPOCH-R.

**Figure 2:**
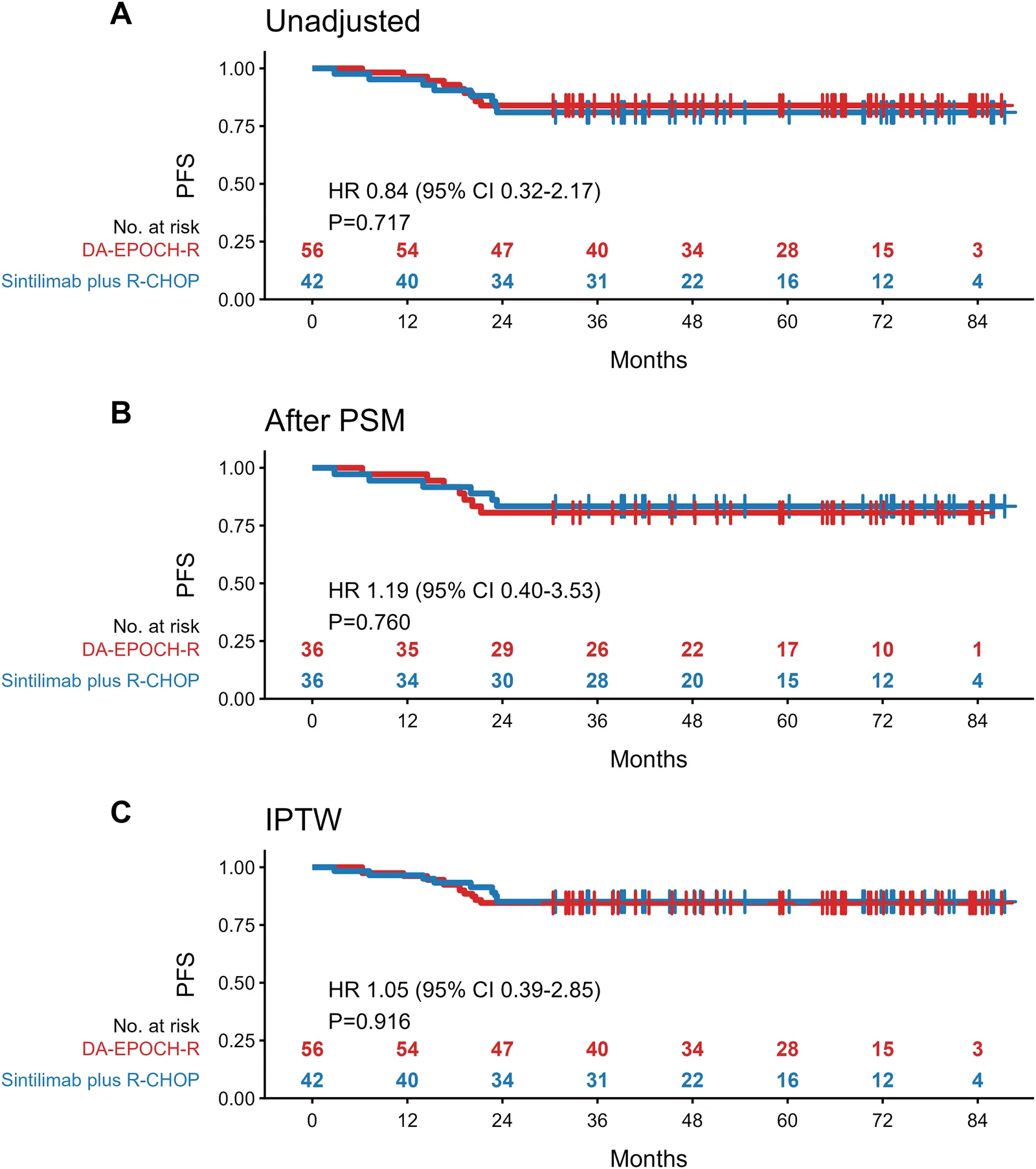
Progression-free survival before matching, after propensity-score matching and after IPTW. **(A)** Kaplan-Meier curves before matching. **(B)** Kaplan-Meier curves after propensity-score matching. **(C)** IPTW-adjusted Kaplan-Meier curves. The full-cohort analysis included 17 PFS events (8 with sintilimab plus R-CHOP and 9 with DA-EPOCH-R), and the matched analysis included 13 PFS events (6 with sintilimab plus R-CHOP and 7 with DA-EPOCH-R). Censoring marks, HRs, P values and risk tables are shown; the IPTW panel displays weighted numbers at risk. HR, hazard ratio; CI, confidence interval; IPTW, inverse probability of treatment weighting; PSM, propensity-score matching.

End-of-treatment Deauville 4–5 uptake was recorded in 6 of 42 patients (14.3%) after sintilimab plus R-CHOP and in 9 of 56 patients (16.1%) after DA-EPOCH-R. Thus, the addition of PD-1 blockade was not associated with a higher rate of residual PET-positive scans in this cohort.

Exploratory analyses addressing residual imbalance were directionally consistent with the primary survival analysis. In a tumor-burden-adjusted Cox model including baseline mass, extranodal involvement and bulky disease, the treatment-associated HR was 0.99 (95% CI 0.38-2.57; P = 0.987). Adjustment for 9p24.1 amplification yielded an HR of 1.28 (95% CI 0.50-3.26; P = 0.608). No treatment-by-IPI interaction was detected (interaction P = 0.852).

### Residual mass

Residual mediastinal mass was frequent after therapy before and after matching. In the matched cohort, any measurable residual lesion was present in 35 (97.2%) patients treated with sintilimab plus R-CHOP and 34 (94.4%) treated with DA-EPOCH-R. Among matched patients who achieved CMR, residual lesion was present in 29 of 30 and 27 of 29 patients, respectively (**Table 2**).

**Table 2:** Residual lesion characteristics before and after propensity-score matching.

Characteristic	Group 1 (n=42)	Group 2 (n=56)	P	SMD	After PSM Group 1 (n=36)	After PSM Group 2 (n=36)	After PSM P	After PSM SMD
Any residual lesion, n (%)	40 (95.2%)	54 (96.4%)	1.000	-0.06	35 (97.2%)	34 (94.4%)	1.000	0.14
Residual lesion among CMR, n/N (%)	34/36 (94.4%)	45/47 (95.7%)	1.000	-0.06	29/30 (96.7%)	27/29 (93.1%)	0.612	0.16
Residual lesion >=2.5 cm, n (%)	12 (28.6%)	13 (23.2%)	0.641	0.12	12 (33.3%)	10 (27.8%)	0.798	0.12

CMR, complete metabolic response; ORR, objective response rate; PMD, progressive metabolic disease; PMR, partial metabolic response; PSM, propensity-score matching; SD, stable disease; SMD, standardized mean difference.

### Safety

The safety profile remained directionally consistent after propensity score matching (**Table 3**). Before matching, grade 3–4 neutropenia occurred in 16 patients (38.1%) in group 1 and 37 patients (66.1%) in group 2 (P = 0.008). After matching, the incidence remained significantly lower in group 1, occurring in 11 patients (30.6%) versus 22 patients (61.1%) in group 2 (P = 0.017), indicating a persistently reduced hematologic toxicity signal associated with the investigational regimen. No statistically significant differences were observed between the two groups in rates of grade 3–4 anemia, thrombocytopenia, febrile neutropenia, or infection after matching. In the DA-EPOCH-R arm, pharmacodynamic dose escalation was achieved in most patients (37/56, 66.1%), arguing against systematic under-dosing as the sole explanation for the observed PFS range.

**Table 3:** Treatment-emergent adverse events before and after propensity-score matching.

Adverse event	Group 1 (n=42)	Group 2 (n=56)	P	SMD	After PSM Group 1 (n=36)	After PSM Group 2 (n=36)	After PSM P	After PSM SMD
Grade 3–4 neutropenia, n (%)	16 (38.1%)	37 (66.1%)	0.008	-0.58	11 (30.6%)	22 (61.1%)	0.017	-0.64
Grade 3–4 anaemia, n (%)	7 (16.7%)	14 (25.0%)	0.456	-0.21	6 (16.7%)	9 (25.0%)	0.563	-0.21
Grade 3–4 thrombocytopenia, n (%)	6 (14.3%)	14 (25.0%)	0.216	-0.27	4 (11.1%)	6 (16.7%)	0.735	-0.16
Febrile neutropenia, n (%)	7 (16.7%)	13 (23.2%)	0.460	-0.16	5 (13.9%)	8 (22.2%)	0.541	-0.22
Grade 3–4 infection, n (%)	4 (9.5%)	11 (19.6%)	0.257	-0.29	4 (11.1%)	8 (22.2%)	0.343	-0.30
Any-grade irAE, n (%)	5 (11.9%)	NA	NA	NA	5 (13.9%)	NA	NA	NA
Grade 3–4 irAE, n (%)	1 (2.4%)	NA			0 (0.0%)	NA		

FN, febrile neutropenia; irAE, immune-related adverse event; NA, not applicable; PSM, propensity-score matching; SMD, standardized mean difference. P values are descriptive and were not adjusted for multiplicity.

Immune-related adverse events (irAEs) were observed only in the group receiving the anti–PD-1 antibody sintilimab. Any-grade irAEs occurred in 5 patients (11.9%) before matching and in 5 patients (13.9%) after matching. Grade 3–4 irAEs were rare, occurring in 1 patient (2.4%) before matching and in none after matching, suggesting an overall manageable immune-related toxicity profile.

### Gene-expression profiling of PMBCL

Gene-expression profiling was available for 75 diagnostic samples, including 31 patients treated with sintilimab plus R-CHOP and 44 treated with DA-EPOCH-R according to the clinical treatment record. Across the molecular subset, 52 of 75 samples (69.3%) carried 9p24.1 (JAK2/PD-L1/PD-L2) amplification, 52 (69.3%) showed high JAK-STAT activation and 45 (60.0%) were classified as immune-escape high. The most frequent coding alterations involved SOCS1, STAT6, CIITA and IL4R, linking cytokine/JAK-STAT signaling to antigen-presentation remodeling. Between treatment groups, 9p24.1 amplification was observed in 20 of 31 (64.5%) versus 32 of 44 (72.7%) samples (P = 0.459), and JAK-STAT-high status in 18 of 31 (58.1%) versus 34 of 44 (77.3%) samples (P = 0.126). PD-L1-high and PD-L2-high distributions were also broadly similar (PD-L1 high, 45.2% versus 54.5%, P = 0.486; PD-L2 high, 54.8% versus 47.7%, P = 0.641), with median PD-L1 log2TPM of 8.17 versus 8.50 (P = 0.094) and median PD-L2 log2TPM of 7.32 versus 7.28 (P = 0.761). These data provide descriptive molecular context and group-level comparability checks; they do not establish predictive biomarkers for treatment selection (**Figure 3****).** *post hoc* descriptive cross-tabulation of 9p24.1 status and PFS events within the 75-sample subset did not show clustering of events in a single treatment–genotype cell. Event numbers in each cell remained in the single digits. No treatment-by-amplification interaction test was therefore performed, and 9p24.1 status is not proposed as a selection biomarker for sintilimab plus R-CHOP in this cohort.

**Figure 3:**
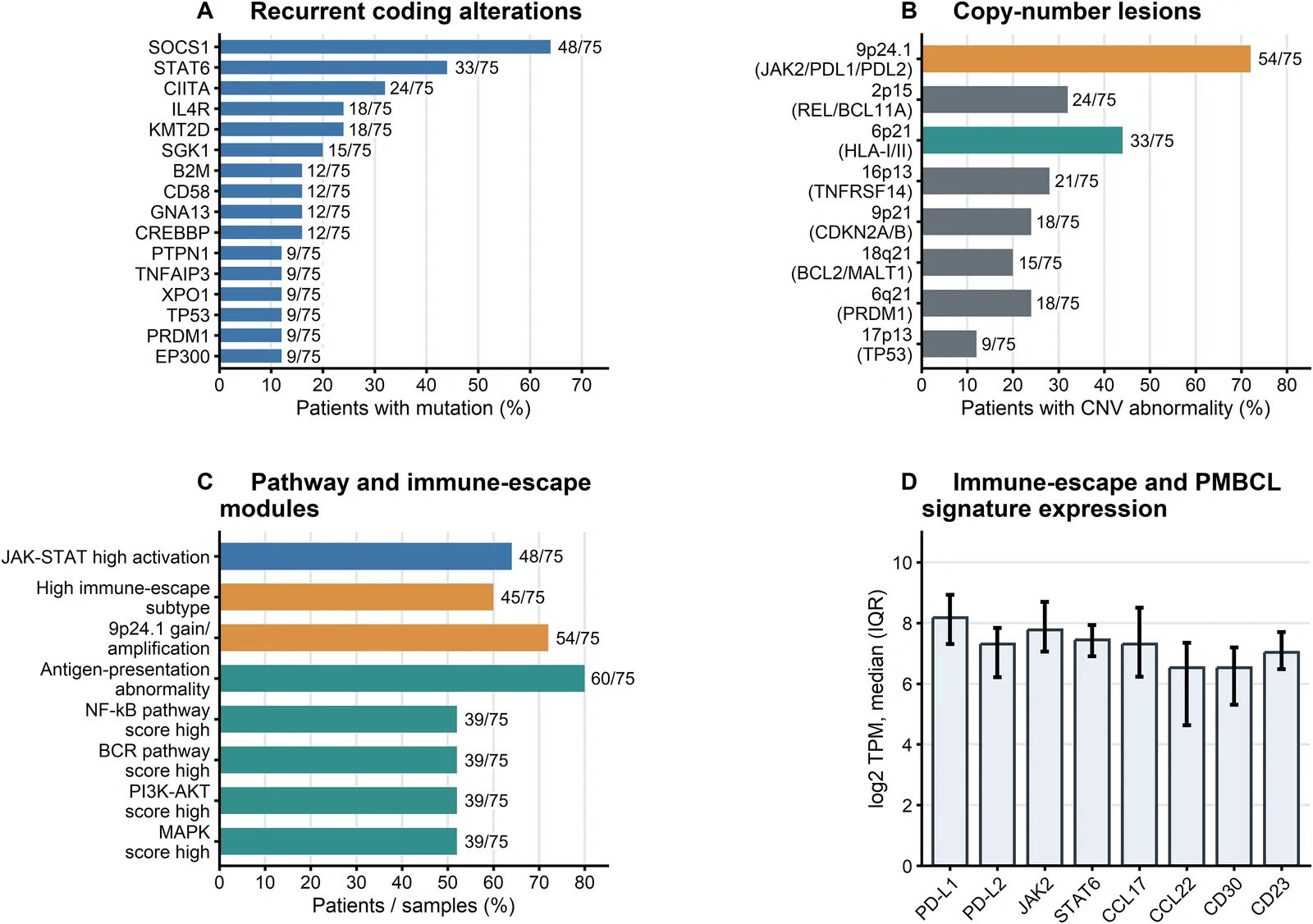
Genomic and transcriptomic immune-escape landscape in PMBCL. **(A)** Recurrent coding alterations in 75 randomly selected profiled diagnostic samples. **(B)** Copy-number abnormalities involving 9p24.1, REL/BCL11A, HLA and other PMBCL-relevant loci. **(C)** Pathway and immune-escape module scores. **(D)** Median log2TPM expression for immune-escape and PMBCL signature genes.

## Discussion

The principal novelty of this study is the real-world description of PD-1 blockade added to R-CHOP as initial therapy for PMBCL, a setting in which randomized evidence is still pending from ANHL1931 (NCT04759586) and ALLG-PACIFIC/NHL35 (20, 21). Primary mediastinal large B-cell lymphoma is defined by a distinctive clinicopathological and molecular profile, including frequent 9p24.1 alterations, JAK-STAT activation and PD-L1/PD-L2 overexpression (1–8). These features provide a compelling biological rationale for integrating PD-1 blockade into chemoimmunotherapy, but the optimal first-line strategy remains unsettled (4, 5, 9–11). In this real-world comparative cohort, we examined whether sintilimab plus R-CHOP could produce overlapping disease-control estimates relative to DA-EPOCH-R while reducing treatment-related hematologic toxicity and informing exploratory PET-directed post-treatment management. This framing is important because the study was not designed to prove equivalence, establish non-inferiority or support radiotherapy omission.

Sintilimab plus R-CHOP showed overlapping response and PFS outcomes to DA-EPOCH-R across unadjusted, propensity-matched and inverse probability-weighted analyses. Survival curves were highly overlapping, without clear early divergence or delayed separation. However, these analyses were based on only 17 PFS events in the full cohort and 13 events after matching, resulting in wide confidence intervals and limited ability to exclude modest treatment effects. A hazard-ratio confidence interval that spans 0.46 to 3.09 in the unadjusted analysis is compatible with both clinically important benefit and clinically important harm; P values greater than 0.05 must not be read as evidence of equivalence. The results should therefore be interpreted as hypothesis-generating rather than as evidence of therapeutic equivalence.

Residual imbalance in clinical and biological variables may still confound treatment-effect estimation despite propensity-score matching and inverse probability weighting. Tumor-burden-related features are particularly relevant in PMBCL. Recent genomic and clinical studies underscore the heterogeneity of PMBCL and the potential importance of molecular context for risk stratification (6, 8, 25, 26). These considerations support cautious interpretation and biomarker-aware prospective validation rather than outcome claims based on retrospective subgroup inference.

From a safety perspective, sintilimab plus R-CHOP was associated with a consistently lower observed burden of grade 3–4 neutropenia compared with DA-EPOCH-R, including after propensity adjustment. This observation is clinically relevant because DA-EPOCH-R requires continuous infusional therapy, dose adjustment and intensive supportive care, and prior real-world studies have highlighted its treatment burden and myelosuppressive toxicity (12–15). Most patients in the DA-EPOCH-R arm underwent dose escalation (66.1%), consistent with delivery of an intensive backbone rather than systematic under-treatment. Nevertheless, the safety comparison remains exploratory because the study was not randomized, multiplicity adjustment was not performed and the matched sample was modest. Substitution of pirarubicin for doxorubicin in both backbones further limits direct toxicity comparisons with doxorubicin-based international series and should be considered when generalizing these safety findings.

A further clinically relevant observation was the high frequency of residual mediastinal masses despite metabolic complete response. This dissociation between anatomical persistence and metabolic remission is consistent with PMBCL biology and with PET-based response literature showing that residual masses after effective therapy do not necessarily represent viable lymphoma (17, 19, 22–24). Importantly, end-of-treatment Deauville 4–5 scans were not more frequent after sintilimab plus R-CHOP than after DA-EPOCH-R, which argues against a dominant inflammatory false-positive PET signal attributable to PD-1 blockade in this cohort. Our findings support PET-guided response evaluation, but they do not provide sufficient evidence to modify radiotherapy decision-making outside prospective clinical trials.

The integrated gene-expression and copy-number analysis confirmed a canonical PMBCL immune-escape signature characterized by frequent 9p24.1 amplification, JAK-STAT pathway activation and PD-L1/PD-L2 expression (6–8, 25, 26). Group-level molecular comparison showed broadly similar 9p24.1 amplification and PD-L1/PD-L2 expression between treatment groups. These data strengthen the biological context for studying PD-1-containing chemoimmunotherapy, but they remain descriptive and cannot be used as predictive biomarkers or as a basis for treatment selection in the current cohort.

This study has several limitations. First, the limited number of progression events reduced statistical robustness and increased uncertainty in hazard-ratio estimation. Second, despite propensity-score matching and inverse probability weighting, residual confounding remains likely because selected clinical and molecular variables were imbalanced. Third, molecular profiling was available only for a subset of patients and was used for group-level comparison rather than molecular-outcome correlation. Fourth, treatment allocation was not randomized, and causal inference should therefore be avoided. Fifth, both regimens used pirarubicin rather than doxorubicin, which affects external generalizability. Finally, the study was conducted in a real-world institutional setting, which supports clinical relevance but may limit generalisability across centres with different PET interpretation, radiotherapy policies and supportive-care practices.

In conclusion, the contribution of this cohort is a real-world signal that frontline PD-1 blockade can be combined with R-CHOP in PMBCL with overlapping PFS estimates versus DA-EPOCH-R and less grade 3–4 neutropenia. These observations do not establish equivalence, non-inferiority, treatment replacement or radiotherapy omission. They provide supportive context for ANHL1931 and ALLG-PACIFIC and justify prospective randomized evaluation with biomarker stratification and standardized PET-adapted response assessment.

## Data Availability

The original contributions presented in the study are included in the article/supplementary material. Further inquiries can be directed to the corresponding author.
